# Duration of the parasitic phase determines subsequent performance in juvenile freshwater pearl mussels (*Margaritifera margaritifera)*

**DOI:** 10.1002/ece3.2740

**Published:** 2017-02-01

**Authors:** Janhavi Marwaha, Knut Helge Jensen, Per Johan Jakobsen, Juergen Geist

**Affiliations:** ^1^ Department of BiologyUniversity of BergenBergenNorway; ^2^ Aquatic Systems Biology UnitTechnical University of MunichFreisingGermany

**Keywords:** coevolution, excystment, fitness, freshwater pearl mussel conservation, host–parasite system

## Abstract

Host–parasite systems have been useful in understanding coevolutionary patterns in sympatric species. Based on the exceptional interaction of the long‐lived and highly host‐specific freshwater pearl mussel (FPM; *Margaritifera margaritifera*) with its much shorter‐lived host fish (*Salmo trutta* or *Salmo salar*), we tested the hypotheses that a longer duration of the parasitic phase increases fitness‐related performance of mussels in their subsequent post parasitic phase, and that temperature is the main factor governing the duration of the parasitic phase. We collected juvenile mussels from naturally and artificially infested fish from eight rivers in Norway. Excysted juvenile mussels were maintained separately for each collection day, under similar temperature and food regimes, for up to 56 days. We recorded size at excystment, post excystment growth, and survival as indicators of juvenile fitness in relation to the duration of the parasitic phase. We also recorded the daily average temperatures for the entire excystment period. We observed strong positive relationships between the length of the parasitic phase and the post parasitic growth rate, size at excystment and post parasitic survival. Temperature was identified as an important factor governing excystment, with higher temperatures decreasing the duration of the parasitic phase. Our results indicate that juvenile mussels with the longest parasitic phase have better resources (larger size and better growth rate) to start their benthic developmental phase and therefore to survive their first winter. Consequently, the parasitic phase is crucial in determining subsequent survival. The temperature dependence of this interaction suggests that climate change may affect the sensitive relationship between endangered FPMs and their fish hosts.

## 1 Introduction

Host–parasite systems have been extensively studied to understand coevolutionary processes. Hosts and parasites are in a continuous arms race against one another and are constantly developing adaptations and counter adaptations against each other. (Dawkins & Krebs, 1979). The survival of a parasite depends on successful infestation of, and establishment on its host. The traditional view is that parasites have a greater evolutionary potential and adaptive plasticity resulting from larger population sizes, higher mutation rates, and shorter generation times compared to their hosts (Ebert, 1994; Gandon & Michalakis, 2002; Kaltz & Shykoff, 1998). In addition to these circumstances, a narrow host range and larger migration rates would most likely result in the parasite being locally adapted to its hosts (Dawkins & Krebs, 1979; Kawecki & Ebert, 2004; Lajeunesse & Forbes, 2002; Morgan, Gandon, & Buckling, 2005). Most studies on host–parasite relationships involve short‐lived parasites, but host–parasite interactions and their effect on parasite fitness are not well investigated in long‐lived parasites. The unionoid freshwater pearl mussel (FPM; *Margaritifera margaritifera*) is one example of a long‐lived specialist parasite, reaching ages of more than 200 years in its northern distribution range. With a generation time that is almost 30 times longer than its host (Geist & Kuehn, 2008), this host–parasite system allows for an interesting study of coevolutionary processes.

The FPM is an endangered bivalve that is listed in IUCN Red List of Threatened Species has been changed to Mollusc Specialist Group^1996^, Annex II and V of the European Habitats and Species Directives (Directive 92/43/EEC 1992) and Appendix III of the Bern Convention (Geist, 2010; Larsen, 2005; Machordom, Araujo, Erpenbeck, & Ramos, 2003; Skinner, Young, & Hastie, 2003). A serious decline of FPM across its geographical range has attracted much concern from national and international conservation organizations (Araujo & Ramos, 2000; Geist, 2010; Machordom et al., 2003; Strayer et al., 2004). Conservation efforts for the species include habitat protection and restoration, release of artificially infested host fish and rearing of juvenile mussels followed by their release into the natural habitat (Bolland, Bracken, Martin, & Lucas, 2010; Gum, Lange, & Geist, 2011; Hastie & Young, 2003; Preston, Keys, & Roberts, 2007; Schmidt & Vandrè, 2010; Ziuganov, Zotin, Nezlin, & Tretiakov, 1994). Rearing programs for the FPM have been put in place in Austria, Belgium, the Czech Republic, Finland, France, Germany, Ireland, Luxembourg, Norway, Spain, and the UK. Current research is focused on understanding the bottlenecks in the life cycle, especially identifying host requirements (Geist & Auerswald, 2007; Geist & Kuehn, 2008; McIvor & Aldridge, 2008; Skinner et al., 2003; Taeubert, Denic, Gum, Lange, & Geist, 2010; Taeubert & Geist, 2017). This knowledge could be useful in improving the understanding of coevolutionary host–parasite interactions as well as in developing improved culturing techniques that can aid conservation.

The complex life cycle of FPM comprises a short‐lived drifting stage (infective glochidia), followed by an obligate parasitic stage on salmonids and a benthic stage during which juvenile mussels remain buried in the river sediment for around 5 years (Bauer, 1987, 1994; Geist, 2010; Hastie & Young, 2003; Moorkens, 1999; Nezlin, Cunjak, Zotin, & Ziuganov, 1994; Smith, 1976; Ziuganov et al., 1994). Although the general life cycle and glochidial larval stages have been described in detail, there are several aspects of parasite–host compatibility, including the influence of the host on the fitness and success of the parasitic (glochidial) and post parasitic (juvenile mussel) stages of the life cycle, which are not well understood (Taeubert & Geist, 2017).

Glochidia, 60–80 μm in size (Moorkens, 1999; Skinner et al., 2003; Wächtler, Dreher‐Mansur, & Richter, 2001), are released by gravid mothers and have to attach to the gills of a suitable fish host, where they become encysted and metamorphose (Araujo, Cámara, & Ramos, 2002; Arey, 1921, 1932a, 1932b; Dodd, Barnhart, Rogers‐Lowery, Fobian, & Dimock, 2005; Geist, 2010; Kat, 1984; Larsen, 2005; Nezlin et al., 1994; Taeubert, Gum, & Geist, 2013; Taeubert et al., 2010; Young & Williams, 1984). This release of glochidia has been reported to be a highly synchronous event with all gravid specimens from each river population releasing their glochidia within a time span of only 1–2 days (Bauer, 1979; Hastie & Young, 2003; Wellmann, 1943; Young & Williams, 1984). The release is typically triggered by abrupt changes in hydrological conditions of the river, causing a change in temperature or water quality parameters (Hastie & Young, 2003; Wellmann, 1943). FPM development and growth is generally dependent on water temperature (Hastie & Young, 2003; Österling, Greenberg, & Arvidsson, 2008; Skinner et al., 2003; Taeubert et al., 2013) and temperature variation can delay reproduction within rivers by several weeks during cold years (Hastie & Young, 2003). However, Hastie and Young (2003) observed several rivers over several years and found glochidial release to be a synchronous event within the river every time. It is, therefore, expected that in rivers located in areas with similar temperature regimes, glochidial release occurs around the same time. Furthermore, once released the glochidia may remain viable for up to 6 days (Jansen, Bauer, & Zahner‐Meike, 2001; Ziuganov et al., 1994). However Young and Williams (1984) observed that the glochidia became lifeless 24 hr post‐release and in natural conditions glochidia only remain in suspension for a short period of time during which they have to infest their host.

In European FPM, glochidia can successfully metamorphose only on the gills of Atlantic salmon (*Salmo salar*), sea trout (*S. trutta f. trutta*) and brown trout (*S. trutta f. fario*) (Geist, 2010; Ieshko et al., 2016; Larsen, 2005; Taeubert et al., 2010, 2013; Young & Williams, 1984). In addition it has been reported that FPM populations exclusively infest either Atlantic salmon or brown trout even if both species are present in the same rivers (Ieshko et al., 2016; Karlsson, Larsen, & Hindar, 2014; Larsen, Hårsaker, Bakken, & Barstad, 2000). The length of the parasitic glochidial developmental phase is highly variable (Ziuganov et al., 1994). In FPM and other species of freshwater mussels, the duration of the host‐dependent phase is expected to be related to either the temperature at which they develop, compatibility with the host, or both (Lefevre & Curtis, 1912; Taeubert, El‐Nobi, & Geist, 2014; Taeubert et al., 2010, 2013; Ziuganov et al., 1994). Two glochidial developmental strategies have been described; one with a developmental period of 20–60 days (Bauer, 1979; Young & Williams, 1984; Ziuganov et al., 1994) and one with a developmental period of 7–9 months (Bauer, 1979; Ziuganov et al., 1994). Both these developmental strategies have been observed within the same mussel population (Ziuganov et al., 1994). In Norway, the long developmental strategy is observed (Larsen, 2005). During the parasitic phase, glochidia grow sixfold–tenfold their original length (Moorkens, 1999; Taeubert et al., 2013) and once they have reached a size larger than 240 μm, all organs of the adult mussel that are required for a benthic existence are present (Ziuganov et al., 1994). Juvenile mussels excyst at sizes between 280–500 μm (Bauer, 1994; Eybe, Thielen, Bohn, & Sures, 2014; Hastie & Young, 2003; Ziuganov et al., 1994; Marwaha, 2012, personal observation).

The length of the excystment period (which starts with the first and ends with the last juvenile mussel dropping off its host) is highly variable (Eybe et al., 2014; Taeubert et al., 2013; Ziuganov et al., 1994) and periods lasting from seven days (Bauer, 1979) up to 148 days (Taeubert et al., 2013) have been reported. We have observed excystment periods from 40 days up to 60 days for Norwegian FPM. The extended excystment period in juvenile mussels is surprising when considering the highly synchronous nature of glochidial release and the short life span of the released glochidia. It would be reasonable to assume that for one FPM population, hosts are infested within a very small time window. We might therefore have expected to see more synchronous excystment as well. Eybe et al. (2014) observed that larger mussels excyst at the end of the excystment period. In addition they also observed that the early excysters had a poor survival, but it remains unclear if this observation from one specific pearl mussel population can be generalized. In order to investigate whether this was a general trend across multiple populations, we used eight Norwegian FPM populations in our experiment. Additionally, we also wanted to observe whether there were any other fitness benefits associated with prolonged excystment.

The objective of this study was to investigate whether the timing of excystment (i.e. the amount of time elapsed since the first mussel excysted) had an effect on the survival and post excystment performance of juvenile pearl mussels from eight Norwegian FPM populations. In particular, we hypothesized that there is a positive correlation between the duration of the FPM parasitic phase on its host with its size and growth during the parasitic phase, but also with beneficial effects on subsequent survival and growth in the post parasitic phase. In addition, we hypothesized that temperature has a strong positive effect on excystment rates. By collecting results from several FPM populations, we would be able to verify whether our hypothesis would hold true as a general trend observed in the FPM life cycle.

## 2 Materials and Methods

In order to test our hypotheses, we used both naturally and artificially infested fish (*S. trutta f. fario* and *S. salar)*. Naturally infested fish were collected from seven rivers (Table 1) in southern Norway by electro‐fishing. The artificial infestations were performed in the river Haukåsvassdraget, where 30 gravid mussels and 100 young of the year farmed trout were kept in a holding tank and natural infestation was allowed to take place. In this case, all glochidial release was synchronous occurring within 2 days. All infested fish, natural or artificial, were transported to the mussel breeding station at Austevoll, Norway, and maintained there until we finished harvesting the juvenile mussels.

**Table 1:** The rivers of origin for each freshwater pearl mussel population, host fish species and number, type of infestation, and the total number of mussels harvested per river population

Mussel river population	Host fish	Number of Fish	Type of infestation	Total mussels harvested
Haukåsvassdraget	*Salmo trutta f. fario*	55	Artificial	353
Hopselva	*Salmo trutta f. fario*	25	Natural	323
Lerangsbekken	*Salmo trutta f. fario*	10	Natural	241
Ereviksbekken	*Salmo trutta f. fario*	31	Natural	237
Steinslandselva	*Salmo salar*	49	Natural	376
Oselva	*Salmo salar*	30	Natural	630
Fossa	*Salmo trutta f. fario*	22	Natural	230
Åreidselva	*Salmo trutta f. fario*	24	Natural	490
			Total	2,880

Water from the lake Kvernavatnet (Austevoll) was used for maintaining fish and juvenile mussels during the experiments. It has a pH of 6.6 and alkalinity of 0.108 mmol/L. Concentrations of aluminum, iron, calcium, magnesium and nitrate were as follows: Al—180 μg/L; Fe—200 μg/L, Ca—4.2 mg/L, Mg—1.8 mg/L, Na—12 mg/L and Nitrate–N—0.15 mg/L. The water was ultraviolet‐light‐treated and filtered through a 30 μm mesh before use. As the water came from the lake, water temperature of the fish holding system followed the natural temperature variation of the lake and was between 5.7 and 17.0°C.

**Figure 1:**
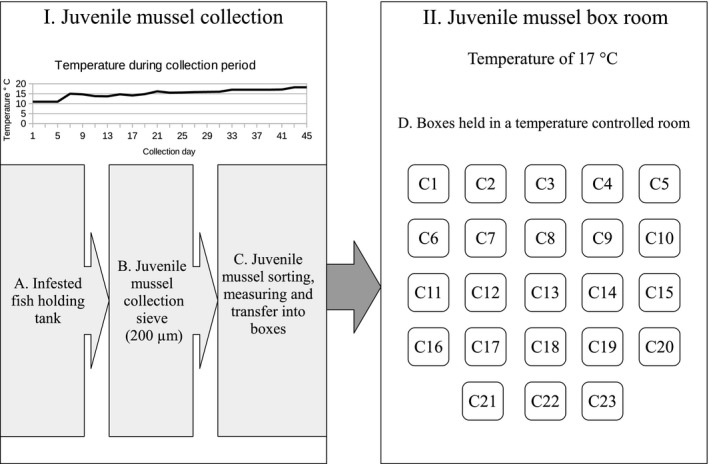
Schematic overview of methods used for each freshwater pearl mussel (FPM) population for a single collection day applied for a total of 24 collection days. Box I: Procedure for juvenile mussel collection. (A) Fish holding tank with infested fish (1 FPM population/tank). (B) Mussel collection sieve (200 μm) from which excysted mussels (end of parasitic phase) were collected every alternate day. (C) Excysted mussels were cleaned, counted and measured (size), and put into boxes (C1–C23) (50 mussels/box). Temperature panel shows the temperature for the different collection days. Box II: (D) Temperature‐controlled mussel box room with boxes from the collection days (C1–C23). Temperature kept constant at 17.00 ± 0.54°C.

Infested fish were transferred and maintained in juvenile mussel collecting chambers until the end of the excystment period, following the methodology originally described by Hruska (1999). All infested fish from a single FPM population were kept in one juvenile mussel collecting chamber. The 200 μm collection sieves were inspected daily to check for the presence of excysted juvenile mussels (Figure 1). Once the excystment of mussels began, the collection sieves were examined every alternate day for the collection of juvenile mussels. Excysted mussels were collected and cleaned thoroughly, that is, only living mussels devoid of all debris (such as fish feces, teeth, scales, and small insects) were put into plastic boxes (175 × 116 × 97 mm; Hruska, 1999). All the mussels from one population from a single collection day were kept separately in boxes (Figure 1). As the number of excysting mussels varied between each collection day (from a minimum of 2 to a maximum of 119), we decided to have an upper limit of 50 mussels per box. This resulted in boxes with different densities of mussels. Although Eybe, Thielen, Bohn, and Sures (2013) observed that mussel density can have an effect on performance, we did not observe such an effect in our experiment (see Section 3). It needs to be noted that Eybe et al. (2013) used much higher densities (200, 300 and 400 mussels per 500 ml box) compared to ours. All boxes were kept in a temperature‐controlled room at a temperature of 17.0 ± 0.54°C (Figure 1). The juvenile mussels were fed every second day with a food mixture described by Eybe et al. (2013). In 10 L of water, we added 1 ml of calcium solution (2.7 mg/L), 250 μl of Shellfish^®^ diet 1800 (Reed Mariculture Inc., Campbell, CA, USA) and 2 ml of a stock solution containing 50 ml of tap water, 0.35 g spirulina (*Arthrospira platensis*) (Bio‐life, Norway), 1 ml Nanno 3600 (Reed Mariculture Inc.) and 10 crushed chironomid larvae (Eybe & Thielen, 2010; Lange personal communication 2012; Scheder, Lerchegger, Jung, Csar, & Gumpinger, 2014). Feeding involved a water change in the box, that is, removal of old food water, rinsing the boxes with clean water before adding 700 ml of food mixture and 100 ml of detritus. The detritus was obtained from a swamp around a small brook, near the breeding station. It was filtered through a 30‐μm sieve and oxygenated for 3 days prior to use.

To investigate whether there was a post excystment fitness effect for juvenile mussels that excysted late, we measured the size at excystment, and post excystment growth rate and survival. For each FPM population, the total number of mussels that excysted on each collection day were counted and measured to the nearest 0.1 mm. The length of each juvenile mussel (defined as the maximum length of the shell at its greatest extension) was measured using a 10× calibrated ocular micrometer in a dissecting microscope. All juvenile mussels were measured on the day of excystment. To compare the growth rates of early and late excysters, juvenile mussels were measured between two time points (using the excystment time point as reference) and average growth rate per day was calculated as the increase in length (μm/day) using the absolute growth rate formula from Hopkins (1992). For assessing survival, we recorded the proportion of surviving juveniles in a given box, from the day of excystment until a given day post excystment. Because mortality is very low after the first week of excystment, we only recorded this endpoint between 22 and 33 days post excystment. Finally, temperature at excystment was recorded to test for links between temperature and number of excysting mussels.

All statistical analyses were computed using the statistical package R version 3.3.2 (R Development Core Team, 2016). To check whether there was a relationship between growth rate and duration of the parasitic phase (i.e. time on gills which was measured as the amount of time passed after the first mussel excysted in a given river), we first established a model with growth rate as a response variable and with the predictors size at excystment and density of mussels. We then used the residuals of this model tested against time on gills. We did this to control for the effect of size and density of mussels. For both models, we used a linear mixed effect modeling (LME) with the river from which each mussel population originated as a random effect factor. To check whether there was a relationship between mean size at excystment and duration of the parasitic phase (time on gills), we used the same type of model (LME) where river was set as a random effect factor. A generalized linear mixed effects model (GLMM) with quasibinomial error term was used to investigate the relationship between the survival during the nonparasitic phase (post excystment) and the duration of the parasitic phase. As in the previous models, the river from which the mussels originated was set as a random effect factor The response variable in this model was the proportion of survivors in a given mussel box until a given post parasitic age ranging from 22–33 days depending on when the boxes were checked for survival. Because survival was not checked at a fixed post parasitic age, we analyzed the data with post parasitic age as a covariate in the model to control for eventual effects of this variable. A GLMM approach with river as a random effect factor was also used to test the relationship between the number of mussels excysting and the temperature. In this model, Poisson was set as an error term as the response variable represents count data. All the above statistical methods are described in Zuur, Ieno, Walker, Saveliev, and Smith (2009).

## 3 Results

**Figure 2:**
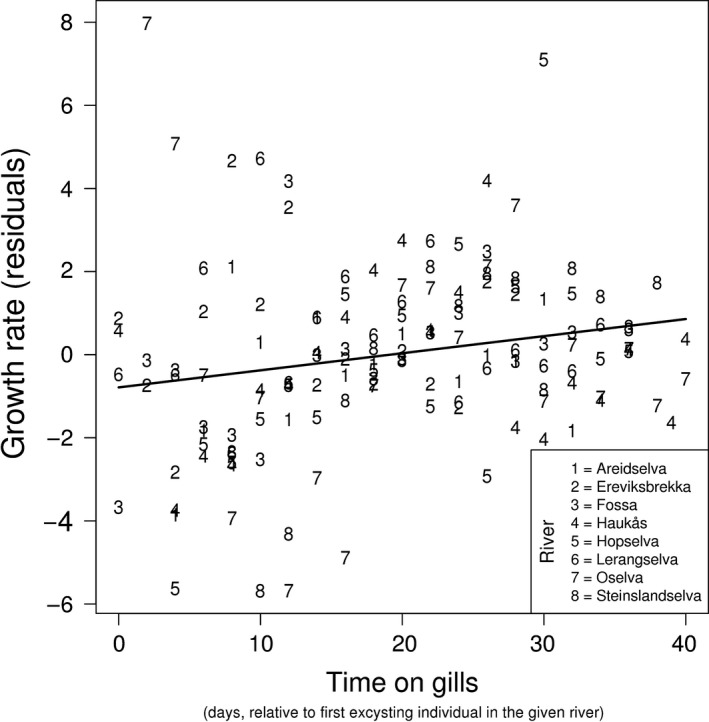
Relationship between time of excystment and residual growth rate (μm/day). The residuals are from a model with size and mussel density as predictors. The line represents model predictions and different symbols indicate different rivers

The duration of the parasitic phase (time on gills) had a positive effect on growth rate (LME: *F*_1,128_ = 5.54, *p*‐value = .02, Figure 2). However, the variability over time on gills was large and there were some individual mussels that dropped off early and at a small size which had higher growth rates compared to those that excysted later and at larger sizes. The model revealed a relatively low effect of individual rivers, where the estimated between river standard deviation was 0.82 and the estimated within river standard deviation was 2.07.

**Figure 3:**
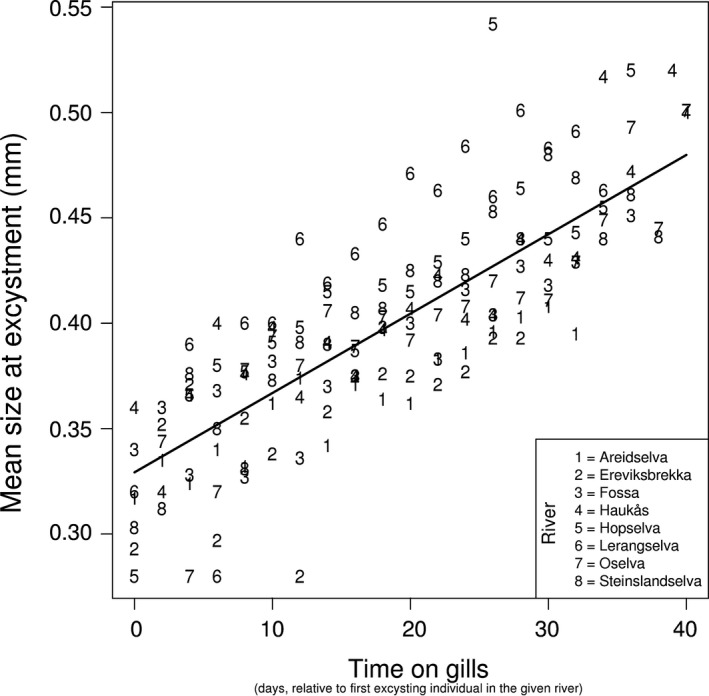
Relationship between the time that mussels spent on the host fish (day 0 refers to the day when excystment started in a given river) and their mean size at excystment

In addition we also observed a positive relationship between the duration of the mussel parasitic phase and their mean size at excystment (LME: *F*_1,137_ = 379.30, *p*‐value < .01, Figure 3). The mussels that dropped off at the end of the excystment period (42 days after the first one excysted) were larger than the first excysters by a factor of 1.49. The estimated between river standard deviation was 0.02, while the estimated within river standard deviation was 0.03.

**Figure 4:**
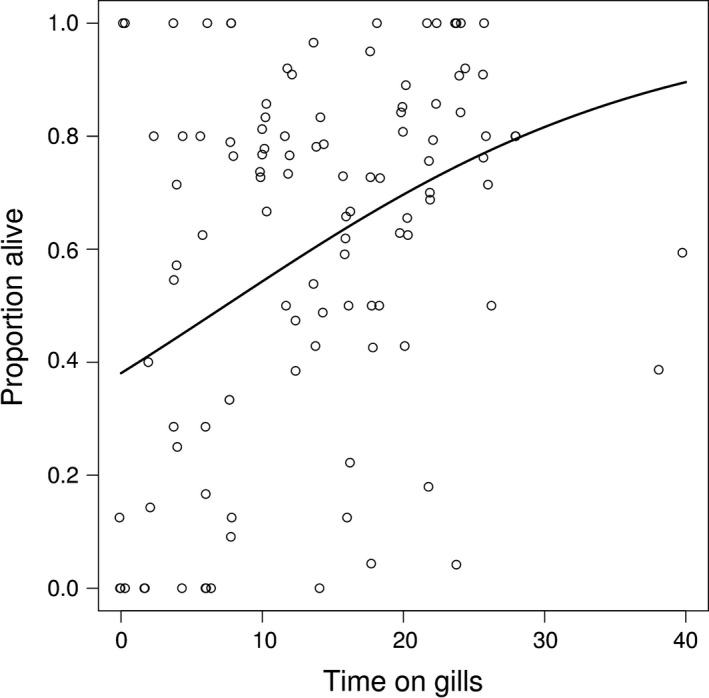
The proportion of survivors depending on the duration of the parasitic phase (time on gills). The line represents model predictions where the covariate (post parasitic age) was set to its mean

The generalized linear mixed effect model used to examine the post parasitic phase survival depending on the duration of the parasitic phase showed a positive relationship between the duration of the parasitic phase (time on gills) and survival (GLMM: *t*‐value = 4.32, *df* = 100, *p*‐value = .02, Figure 4). The estimated between river standard deviation was 0.41, while the estimated within river standard deviation was 0.59.

**Figure 5:**
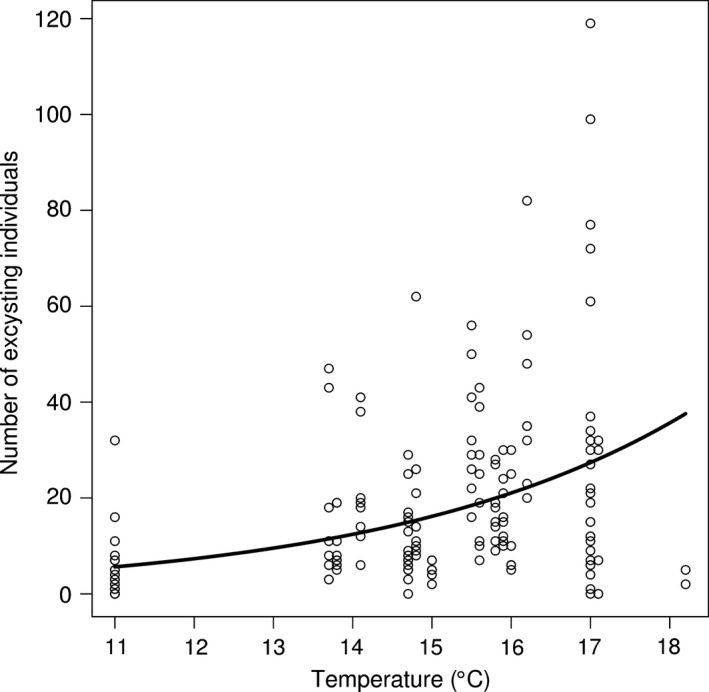
Number of excysting individuals depending on temperature

There was a positive relationship between temperature and the number of mussels that excysted (GLMM: *df* = 152, *t*‐value = 6.05, *p*‐value < .01, Figure 5) where the predicted number of excysted individuals at 11 and 18°C was 5.63 and 35.65 individuals, respectively. The estimated between river standard deviation was 0.33, while the estimated within river standard deviation was 3.43.

## 4 Discussion

The results of this study suggest that the duration of the parasitic phase of FPM larvae on their fish hosts has positive effects on their subsequent size and growth rates. In addition juvenile mussels with a longer parasitic phase had higher survival rates. Moreover, and in line with previous studies (Taeubert et al., 2013), temperature was identified as an important driver governing the numbers of dropped‐off juveniles. All the eight FPM populations that were investigated consistently showed these results.

In the case of the naturally infested fish, results maybe confounded due to the asynchronous release of glochidia. However, this appears highly unlikely based on evidence from the literature and our observations of a highly synchronous release over several years for the populations under study (data not shown). We have also had parts of these FPM populations at the rearing facility in subsequent years and have observed that all glochidial releases occurred synchronously, within a period of 1–2 days. In addition several authors have also observed a similar release of glochidia (Bauer, 1979; Wellmann, 1943; Young & Williams, 1984). Hastie and Young (2003) also observed this behavior over several years. Furthermore, all the FPM populations used in our experiment were from rivers in southern parts of Norway which have similar geographical, hydrological, and temperature conditions.

Mussels that excysted later during the excystment period had clearly benefited in terms of size, post excystment growth and survival. Late excysters will most probably have better resources to start their benthic existence and hence have better survival (Eybe et al., 2014; Österling & Larson, 2013). This would be particularly important during the first winter, especially in Norway and other areas with colder climatic conditions where winter temperatures are lower compared to central or southern Europe. Our results are in line with the practical observation that juvenile mussel survival during the first winter depends on the mussels attaining a critical shell length of 1 mm in order to survive it (Gum et al., 2011; Lange & Selheim, 2011).

The difference in fitness between the early and late excysters could be due to a variable developmental speed of the glochidia which in turn could be related to parasite–host compatibility. In a FPM‐host suitability experiment, Taeubert et al. (2010) observed that the most suitable fish strain had higher infestation rates as well as highest glochidial growth rates. They also observed that glochidial sizes were highly different among individuals of the same host species/strain. They suggested that this was due to the differences in compatibility between the parasite and host. Parasite–host compatibility will influence the successful encystment of the glochidia, which is essential for a successful parasitic phase (Haag, 2012; Taeubert & Geist, 2017). When glochidia attach to the gills of the specific host, they elicit an immune response and are then encysted by the fish host. However, those that cannot elicit an immune response from the fish host are not encysted and are shed off (Nezlin et al., 1994). On attaching to an unsuitable host an “abnormal” cyst forms which leads to sloughing off or death of the glochidia (Rogers‐Lowery & Dimock, 2006). The cyst is essential for the parasitic phase (Haag, 2012) because it is thought to provide nutrition and mechanical protection to the glochidia (Arey, 1932b, 1932c; Wächtler et al., 2001; Ziuganov et al., 1994). Thus, it is likely that the degree of compatibility with the host fish will influence how successfully the host builds the “house” cyst around the glochidia, which in turn affects the establishment and degree of nutrition available to the developing glochidia. We believe that this parasite–host compatibility could be related to the major histocompatibility complex (MHC) variability of the fish hosts. It has been shown that MHC variability influences growth of parasites (Kurtz et al., 2004). Furthermore, we have observed (Marwaha et al., 2014 unpublished data) that juvenile mussels were larger on MHC heterozygous fish compared to MHC homozygous fish. Thus it is very likely that the success of glochidial encystment, and therefore growth and development, depends on the MHC variability of the fish hosts.

Other factors could also influence the availability of nutrition to the developing glochidia (Taeubert et al., 2013). For example, the position of the cyst on the gills of the host fish might be important. Glochidia encysted on the gill rakers could have different nutrition available compared to those on the gill filaments. In turn, this could influence developmental speed (Taeubert et al., 2013).

The lower survival we observed in juvenile mussels with a short parasitic phase is most probably related to premature excystment (Eybe et al., 2014). Eybe et al. (2014) proposed that mussels, while still encysted, continue to grow during the excystment period by continuously taking up nutrients from their host. Premature excystment could result in small, poorly developed mussels that are unable to survive the first month in their benthic habitat.

In line with other reports (Taeubert et al., 2013), we also observed that temperature was an important environmental cue for excystment. There is likely an optimal time for excystment of mussels in relation to water temperature, that is, at the ideal temperature the maximum numbers of mussels will excyst. Buddensiek (1995) observed that juvenile mussel growth was restricted to the warmer months of the year and they stopped growing in the cold winter months, a pattern that results in tree‐ring like growth structures in the mussel shells (Geist, Auerswald, & Boom, 2005). Therefore, it would be beneficial for a mussel to excyst at a temperature at which the juvenile mussels can start their benthic stage under ideal conditions and benefit from maximum growth before the winter period.

With the development and growth of FPM being dependent on water temperature (Hastie & Young, 2003; Österling et al., 2008; Skinner et al., 2003; Taeubert et al., 2013), variation in temperature can influence glochidial metamorphosis (Hruska, 1992; McIvor & Aldridge, 2008), growth (Larsen, 2005), duration of the parasitic phase and release of glochidia from their cysts (Eybe et al., 2014; Hruska, 1992; Larsen, 2005; Lefevre & Curtis, 1912; McIvor & Aldridge, 2008 Ziuganov et al., 1994). Reproduction stages of FPM are thought to depend on either a critical minimum water temperature or a summation effect (“minimum number of cumulative day‐degrees”) or both these factors (Hastie & Young, 2003; Jungbluth & Lehmann, 1976). Thus any change in the natural temperature regime (e.g. due to climate change) can affect the sensitive relationship between parasite and host which is particularly crucial in the context of conservation of the endangered FPM. Although our data suggest that temperature appears to be the most important factor which influences the glochidial development and timing of the start of excystment, it does not explain why the post excystment growth, under equal temperature conditions, is higher in those mussels that excyst late. This observation can only be explained by other factors such as the previously discussed parasite–host compatibility.

Some mussel populations have prolonged excystment periods. This could be advantageous, as it allows for the dispersal of juvenile mussels over a larger river area through host migration (Taeubert et al., 2013; Watters & O'Dee, 1999). A good location in the river would improve chances of survival and reduce competition for nutrients (Taeubert et al., 2013). However, the longer the mussels stay on their host, the probability that the host dies or gets eaten increases. At the same time, if multiple mussels all drop in the same spot, there could be an increased risk of predation and intraspecific competition. A prolonged excystment period can be seen as a strategy to reduce risk by bet hedging.

## 5 Conclusions

Our results strongly indicate that the duration of the parasitic phase of FPM has a significant effect on their post excystment performance. We found that juvenile mussels with the longest parasitic phase had a size, growth rate, and survival advantage over those with the shortest one. Our results imply that post excystment fitness (performance) of the juvenile mussels most likely depends on parasite–host compatibility, and that temperature changes, for example due to climate change, can potentially affect the sensitive balance in this host–parasite interaction. Further research will allow us to identify the exact underlying factors that govern parasite–host compatibility.

## Conflict of interest

None declared.

## Data Accessibility

The data set used in this study will be available at Dryad Digital Repository (DOI: doi:10.5061/dryad.3nb53).
